# Chromosome-scale genome assembly and annotation of Huzhang (*Reynoutria japonica*)

**DOI:** 10.1038/s41597-025-04773-8

**Published:** 2025-03-21

**Authors:** Jumei Zhang, Qing Xu, Lei You, Bin Li, Zezhi Zhang, Wenyao Lin, Xiangyin Luo, Zhengxiu Ye, Lanlan Zheng, Chen Li, Junpeng Niu, Guodong Wang, Honghong Hu, Chao Zhou, Yonghong Zhang

**Affiliations:** 1 National Key Laboratory of Crop Genetic Improvement, Hubei Hongshan Laboratory, Huazhong Agricultural University, Wuhan, 430070 China; 2 Key Laboratory of Three Gorges Regional Plant Genetics and Germplasm Enhancement (CTGU)/Hubei Key Laboratory of Tumor Microenvironment and Immunotherapy, College of Biological and Pharmaceutical Sciences/College of Basic Medical Science, China Three Gorges University, Yichang, 443002 China; 3 Shiyan Key Laboratory of Medicinal Plants and Evolutionary Genetics, Hubei Key Laboratory of Wudang local Chinese Medicine Research, School of Basic Medical Sciences, Hubei University of Medicine, Shiyan, 442000 China; 4 Shiyan Academy of Agricultural Sciences, Shiyan, 442000 China; 5 College of Life Sciences, Key Laboratory of Medicinal Resources and Natural Pharmaceutical Chemistry of Ministry of Education, Engineering Research Center of High Value Utilization of Western China Fruit Resources of Ministry of Education, Xi’an, 710119 China

**Keywords:** Plant genetics, Genome

## Abstract

*Reynoutria japonica*, commonly known as Huzhang or Japanese knotweed, is a perennial herbaceous plant belonging to the family *Polygonaceae* and order *Caryophyllales*. This plant is valued for its traditional medicinal uses in China. In this study, we present a high-quality, chromosome-scale reference assembly for *R. japonica* using a combination of PacBio long-read sequencing, Hi-C reads, and Illumina short-read sequencing. The final assembled genome spans approximately 3.30 Gb, with a contig N50 of 1.39 Mb. Notably, 99.22% of the assembled sequences were anchored to 22 pseudo-chromosomes, and 74.79% of the genome is composed of repetitive elements. Genome annotation revealed 68,646 protein-coding genes and 14,788 non-coding RNAs. This genomic resource provides a robust foundation for comparative genomics and will enable deep insights into the evolutionary relationships across related species.

## Background & Summary

*Reynoutria japonica*, also known as *Polygonum cuspidatum*, and commonly referred to Huzhang in Chinese and Japanese knotweed in Japan^1^, is a perennial herbaceous species belonging to the family Polygonaceae. In Europe, it is recognized as one of the most invasive alien plant species and is currently prevalent across nearly all European countries^2^. However, in the Qinba mountain region of central China, it is valued as a traditional medicinal plant and a vegetable crop^3^. Over the past five years, rapid advancements in sequencing technologies have significantly expanded our understanding of the complete genomes of medicinal plants^4^. To date, the genomes of approximately 126 key Chinese herbs have been described^4^, including *Artemisia argyi*^5^, *Dendrobium officinale*^6^, *Taxus wallichiana*^7^, *Coptis chinensis*^8,9^, and *Andrographis paniculata*^10^. Among these efforts, a previous study utilizing next-generation short-read Illumina sequencing and transcriptome-assisted annotation produced a draft genome assembly for *P. cuspidatum*, revealing a genome size of 2.56 Gb and identifying 55,075 functional genes^11^. Due to the high abundance of transposable elements (TEs) in the *R. japonica* genome, however, this draft genome remains incomplete^11^, hindered by technological limitations inherent to the sequencing platform. These TEs complicates *de novo* assembly, leading to numerous gaps and errors, particularly in complex genomic regions. These challenges emphasize the necessity of further refinement to achieve a more accurate and complete genome representation in *R. japonica*.

In this study, to overcome the difficulties associated with assembling the *R. japonica* genome, we employed a combination of Illumina sequencing, high-throughput chromosome conformation capture (Hi-C) sequencing, and single molecule real-time (SMRT) sequencing. Subsequently, the completeness and contiguity of the assembled genome were evaluated. The final assembled genome spans approximately 3.30 Gb with a contig N50 of 1.39 Mb. 99.22% of the assembled sequences were anchored to 22 pseudo-chromosomes, and 74.79% of the genome consisting of repeat elements. Genome annotation revealed 68,646 protein-coding genes and 14,788 non-coding RNAs. The present high-resolution genome of *R. japonica* provides a valuable reference for the entire *Polygonum* genus, offering insights into comparative genomics and advancing our understanding of evolutionary relationships and gene functions across closely related species.

## Methods

### Sample collection and DNA/RNA extraction

**Fig. 1:**
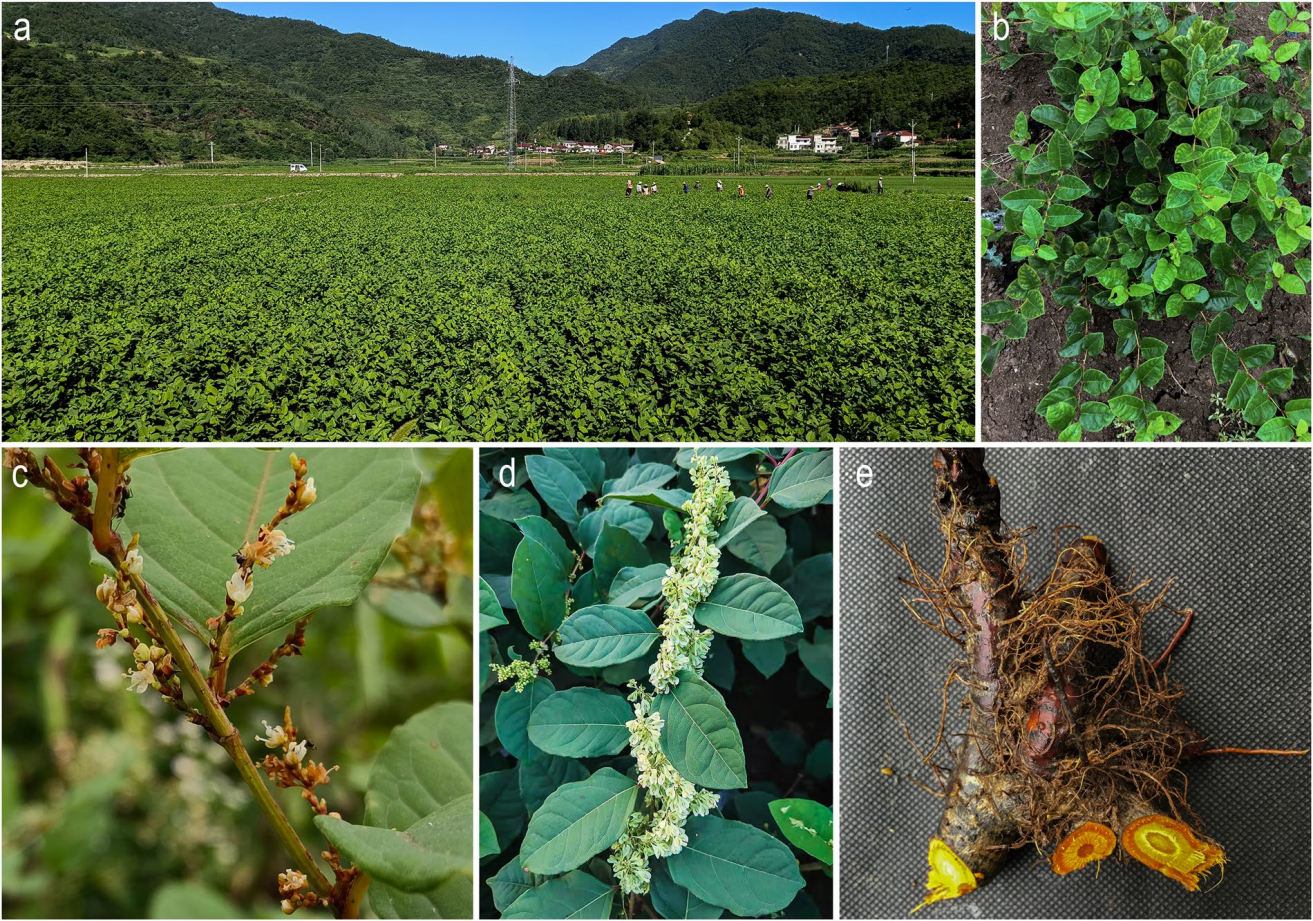
Photographs taken from the sampled plant of *Reynoutria japonica*. (**a**) Cultivation field; (**b**) One-year-old plants of *P. japonica*; (**c**) Inflorescences with floral buds; (**d**) Fruits; (**e**) Underground tubers.

*R. japonica* plants were cultivated in the Qinba Mountains of Shiyan, Hubei Province, China^12^. Fresh young leaves from one-year-old plants were harvested and immediately frozen in liquid nitrogen (Fig. 1a,b). Genomic DNA was extracted using an improved CTAB method^13^. Five tissues types (leaves, stems, flowers, roots, and fruits) were collected from a single individual for RNA extraction (Fig. 1c–e). The samples were promptly frozen in liquid nitrogen and stored at −80 °C until extraction. Total RNA was extracted using the TruSeq Stranded mRNA preparation kit, according to the manufacturer’s instructions.

### Genome sequencing

The sequencing library (DNBSEQ) was constructed and detected by MGIEasy Universal DNA Library Prep Set (MGI), Qubit™ dsDNA BR Assay Kit (Invitrogen) and Qubit® ssDNA Assay Kit (Invitrogen), and the sequencing was conducted on the MGISEQ-2000, generating 150-bp paired-end reads (PE150). For PacBio sequencing, DNA libraries were prepared using the SMRTbell® prep kit 2.0, following the manufacturer’s instructions. Sequencing was performed on the PacBio Sequel II platform. For Hi-C sequencing, DNA was purified using the QIAamp DNA Mini Kit (CAT#51306, Qiagen) according to the manufacturer’s protocol. The Hi-C library was subsequently sequenced on the MGISEQ-2000 platform. All genome sequencing and Hi-C sequencing data were derived from a single plant. Details of the data from each platform are provided in Table 1. Raw reads from transcriptome sequencing were processed sequenced using the Illumina NovaSeq. 6000 platform to generate 5.24–6.91 Gb of paired-end reads. These transcriptomic data were utilized for subsequent gene structure annotation.

**Table 1:** Data Output Statistics for Genome Sequencing.

	Total reads	Total bases(bp)	Clean reads	Clean bases(bp)	Q20 rate (%)	Q30 rate (%)	GC (%)
Illumina	1,840,965,242	276,144,786,300	1,830,267,106	274,348,449,468	96.18%	88.13%	37.54%
HiC	2,149,087,024	322,363,053,600	2,128,053,738	319,123,525,782	92.57%	79.59%	38.54%
PicBio	—	—	13,307,208	252,451,042,968	—	—	39.60%

In total, we generated 276.14 Gb (~84 × coverage) Illumina reads with a Q20 rate at 96.18%, 322.36 Gb (~98 × coverage) Hi-C reads with a Q20 rate at 92.57%, 252.45 Gb (~77 × coverage) PacBio reads, and 123.30 Gb RNA data with GC content was stable at 39.60%. These controls ensure the reliability of our sequencing data (Table 1).

### Genomic survey

**Fig. 2:**
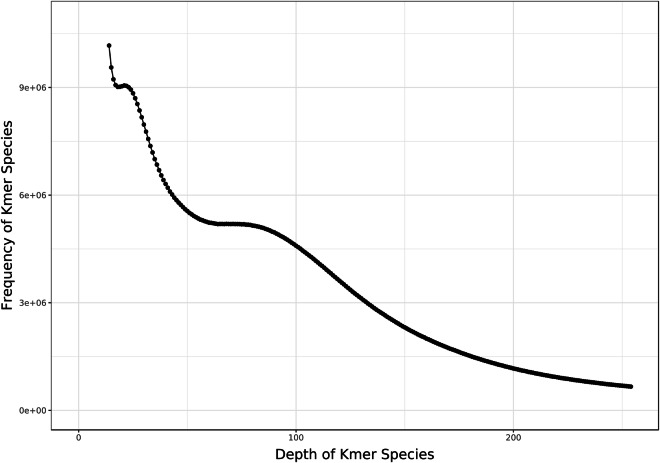
Frequency distribution of depth and K-mer Species.

The generated Illumina sequencing data were processed using Fastp software (v0.23.3)^14^ with default parameters. This process included discarding reads with adapter contamination, trimming low-quality bases from both the 5′ and 3′ ends using a sliding window approach, and correcting mismatched base pairs in the overlapping regions of paired-end reads. Then the clean data were then used for K-mer analysis with GCE software (v1.0.2)^15^. Based on the 17-mer distribution (Fig. 2), information on the peak depth (86) and the number of 17-mers (241,952,873,067) was obtained and used to estimate genome size (2,813 Mb) (Table 2). The estimation was carried out using the following formula: Genome size = K-mer num/Peak depth^16^. Additionally, based on K-mer analysis, the heterozygosity rate (0.35%) and proportion of repeat sequence (81.28%) were calculated according to the methods described by Liu *et al*.^16^.

**Table 2:** Genome survey statistics for *Reynoutria japonica*.

K-mer number	K-mer Depth	Genome Size (Mb)	Heterozygous Ratio (%)	Repeat (%)	GC content (%)
241,952,873,067	86	2,813	0.28	76.25	37.5

### Genome assembly and quality assessment

**Fig. 3:**
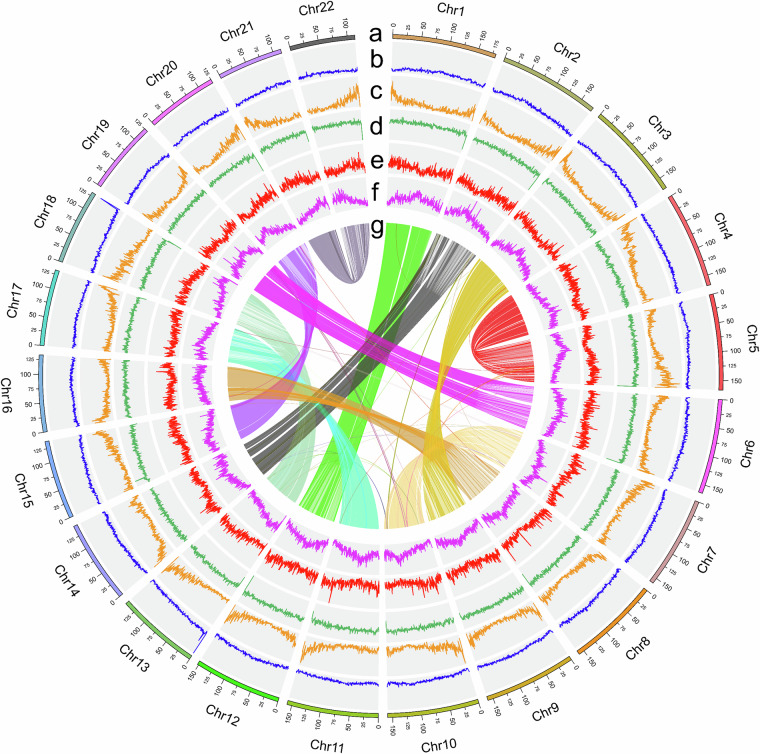
The circos diagram of *P. japonica* genome. Circles (**a**) to (**g**) represent 22 pseudochromosomes of the *P. japonica* pseudochromosomes (**a**), GC content (**b**), gene density (**c**), repeat density (**d**), copia elements density (**e**), gypsy elements density (**f**) and collinearity (**g**) between the pseudochromosomes (**g**), respectively. All calculations were done within 1 Mb windows.

**Fig. 4:**
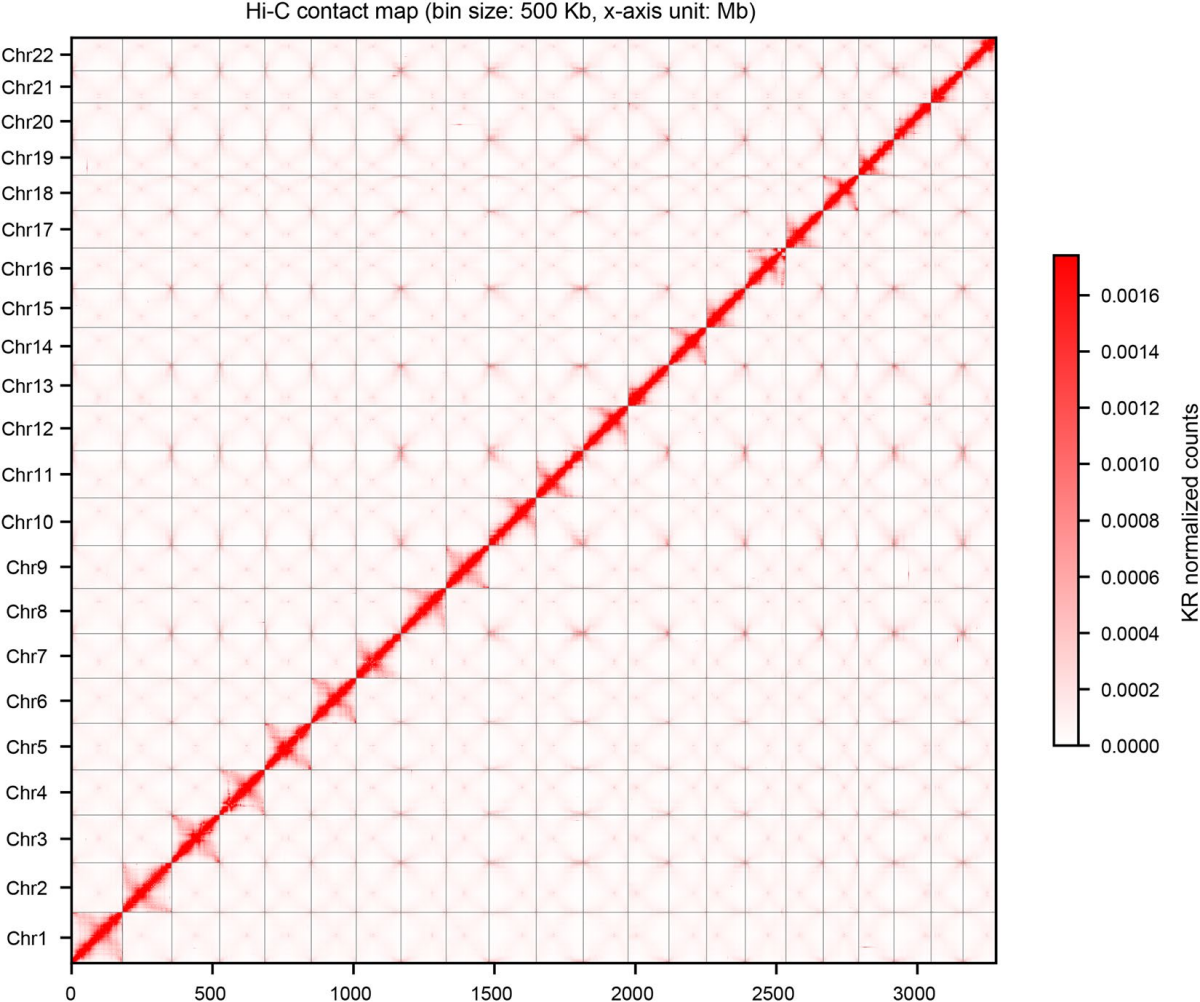
Hi-C interactive heatmap (bin size = 100 kb). Genome-wide chromatin interactions in the *R. japonica* genome at 100-kb resolution. Color blocks represent interaction strength, ranging from white (low) to red (high).

The 252.45 Gb PacBio subreads were initially assembled using Canu v2.1.1^17^. The primary assembled genome was polished using PacBio long reads processed with Arrow (Arrow: https://github.com/PacificBiosciences/GenomicConsensus) and short reads processed with Pilon^18^ with default parameters. Based on this primary genome assembly, Hi-C short reads were subsequently employed to construct chromosomes for elongate loach. Approximately 84,647,123 of valid paired reads, filtered from the total pool of 331.31 Gb of clean Hi-C reads (Table 1), were utilized for assembly and error correction in scaffold extension and chromosome assembly. Quality control measures were applied to the Hi-C reads using Juicer (v1.6)^19^. The contig assembly was subsequently organized into a chromatin scaffold utilizing 3D-DNA^20^ (v180922, parameter -r2). The visualization of Hi-C interactions was conducted with 3D-DNA and further examined through Juicebox (v1.11.08)^21^. The *de novo* genome assembly generated a draft genome of approximately 3,297.29 Mb, consisting of 9,085 contigs with an N50 of about 1.39 Mb and a scaffold N50 of roughly 158.33 Mb (Table 3). Finally, a total of 22 pseudochromosomes were obtained (Figs. 3 and 4), encompassing 99.22% (3,271.86 Mb) of the assembled contigs (Table 3). The GC content of these pseudochromosomes was approximately 38.40% (Table 3), ranging from 38.10% to 38.58% (Table 4).

**Table 3:** Features of the *R. japonica* genome assembly and annotation.

Assembly characteristics	Values
Genome size (bp)	3,297,286,268
The number of Contigs	9,085
Contig N50 (bp)	1,385,282
Contig N90 (bp)	115,017
The number of Scaffolds	466
Scaffold N50 (bp)	158,325,324
Scaffold N90 (bp)	125,097,835
The number of Chromosomes	22
Chromosome length (bp)	3,271,862,532
Anchored rate of bases to the pseudochromosomes	99.22%
GC content	38.40%
Number of annotated genes	68,646

**Table 4:** Summary of the structure of 22 pseudochromosomes.

Pseudo-chromosome ID	Sequences length (bp)	GC content
Chr1	180,684,670	38.26%
Chr2	174,646,997	38.33%
Chr3	169,466,872	38.29%
Chr4	167,496,457	38.11%
Chr5	166,575,596	38.12%
Chr6	164,655,792	38.35%
Chr7	159,929,468	38.16%
Chr8	159,732,238	38.31%
Chr9	159,383,744	38.13%
Chr10	158,325,324	38.23%
Chr11	158,284,252	38.26%
Chr12	152,074,154	38.26%
Chr13	144,989,421	38.58%
Chr14	143,924,633	38.28%
Chr15	137,425,164	38.31%
Chr16	133,364,212	38.31%
Chr17	132,324,119	38.29%
Chr18	131,086,629	38.38%
Chr19	125,495,850	38.20%
Chr20	125,097,835	38.28%
Chr21	113,832,260	38.10%
Chr22	113,066,845	38.15%
Uplaced	25,423,736	47.00%

The assessment of genome assembly’s completeness was conducted using the Benchmarking Universal Single-Copy Orthologs (BUSCO v5.4.3) assessment^22^. After searching against the eudicots_odb10 database, *R. japonica* genome was validated to 95.20% of 2,326 BUSCO groups (Table 5). These findings collectively demonstrate the high sequence integrity, continuity, and accuracy of the *R. japonica* assembly, meeting reference-quality standards.

**Table 5:** BUSCO assessment result.

	Genome evaluation in BUSCO	Gene set evaluation in BUSCO
Complete BUSCOs (C)	95.20%	94.30%
Complete and single-copy BUSCOs (S)	19.20%	22.80%
Complete and duplicated BUSCOs (D)	76.00%	71.50%
Fragmented BUSCOs (F)	1.30%	0.60%
Missing BUSCOs (M)	3.50%	5.10%
Total BUSCO groups searched	2,326	2,326

### Repeat annotation

A combination strategy of homology-based and de novo prediction methods was used to identify the repeat elements (REs) in the *R. japonica* genome. In the homology-based approach, RepeatMasker v4.0.6 (-e rmblast) and RepeatProteinMask v4.0.6 (-pvalue 0.0001)^23^ were employed to identify repeats at both the DNA and protein levels by searching against the RepBase library^24^ and the TE protein database. Tandem repeats were characterized using Tandem Repeats Finder (TRF, v4.07)^25^. Additionally, LTR_FINDER v1.0.6^26^ with default parameters were utilized for the *de novo* prediction of novel repetitive elements.

In this study, the annotated 2,465.7 Mb of repetitive sequences accounted for 74.79% of the assembled *R. japonica* genome (Table 6). Among these sequences, Long Terminal Repeats (LTR) constituted the greatest proportion (47.918%, consist of 6.831% Copia, 16.958% Gypsy, and 24.129% Other LTRs), followed by DNA transposons (DNA) (3.750%), Long Interspersed Nuclear Elements (LINE) (2.707%), and Short Interspersed Nuclear Elements (SINE) (0.004%) (Table 6). The repetitive regions of the genome were then masked before proceeding with further gene prediction.

**Table 6:** Classification of repeat annotation in *R. japonica*.

Type		Count	bpMasked	%masked
DNA		361,452	123,655,557	3.750%
LINE		179,811	89,270,298	2.707%
LTR	Gypsy	784,044	559,129,203	16.958%
	Copia	298,684	225,219,958	6.831%
	Others	1,141,149	795,573,075	24.129%
	Total LTR	2,223,877	1,579,922,236	47.918%
SINE		1,432	127,148	0.004%
Simple_repeat		911,757	26,630,451	0.808%
Satellite		911,757	1,036,954	0.031%
Unknown		3,132,445	1,415,727,046	42.938%
Total		6,009,012	2,465,775,058	74.785%

### Non-coding gene annotation

In this study, we examined the gene structures of tRNAs, rRNAs, and other non-coding RNAs. tRNAs were predicted using the t-RNAscan-SE v1.4^27^ program (http://lowelab.ucsc.edu/tRNAscan-SE/). Given the high conservation of rRNAs, we chose reference rRNA sequences from closely related species and used BLAST (blastn, evalue 1e-05) for rRNA sequences prediction. We also identified additional ncRNAs such as miRNAs and snRNAs by searching the Rfam^28^ database with Infernal v1.1^29^ using default parameters. This analysis result in the annotation of 14,788 noncoding genes, which include 339 miRNAs, 7,508 tRNAs, 1355 rRNAs, and 5,586 snRNAs (Table 7).

**Table 7:** Statistics of noncoding genes.

Categories		Number	Average length (bp)	Total length (bp)	Proportion (%)
miRNA		339	130.95	44,391	0.001
tRNA		7,508	74.46	559,016	0.017
rRNA	18S	212	1,807.82	383,258	0.012
	28S	301	3,716.06	1,118,535	0.034
	5.8S	304	153.60	46,693	0.001
	5S	538	111.52	60,000	0.002
	Total rRNA	1,355	5,789	1,608,486	0.049
snRNA	CD-box	5,064	104.95	531,448	0.016
	HACA-box	174	122.25	21,271	0.001
	splicing	348	138.99	48,369	0.001
	Total snRNA	5,586	366.19	601,088	0.018

### Protein-coding genes prediction and functional annotation

To ensure precise gene prediction, a comprehensive approach combining *de novo* prediction, homology-based prediction, and transcriptome-based prediction. First, it predicted the *de novo* gene structure with AUGUSTUS v3.2.1^30^ and GlimmerHMM v.3.0.4^31^. Second, homologous protein sequences of three other plants in the *Caryophyllales* order, including *Fagopyrum tataricum*, *Beta vulgaris*, and *Spinacia oleracea* obtained from NCBI were aligned with the *R. japonica* genome with TBLASTN. Third, the RNA-seq data from five tissues were mapped onto the assembled genomes with HISAT2 v.2.2.178^32^. RNA-seq data were filtered using SOAPnuke software (v2.1.0)^33^ with the following parameters: -lowQual = 20, -nRate = 0.005, and -qualRate = 0.5. The data were processed by removing paired reads containing adapters, discarding those with more than 0.5% Ns, and eliminating low-quality reads where over 50% of bases had a quality score (Q) ≤ 20. Subsequently, StringTie v.2.1.679^34^ identified potential exon regions, and ORFs were predicted via TransDecoder v.5.1.0 using the transcript sequences. Finally, the gene sets were integrated by braker v2.1.5^35^.

In this study, we identified 68,646 protein-coding genes in the *R. japonica* genome. The gene structure and gene elements, including average transcript length, average CDS length, and average exon and intron length, were compared with the above three related species in the order *Caryophyllales* (Table 8).

**Table 8:** Comparative analysis of gene elements.

Species	Number	Average transcript length (bp)	Average CDS length (bp)	Average exons per gene	Average exon length (bp)
*Reynoutria japonica*	68,646	4,418.33	214.72	13.95	290.37
*Fagopyrum tataricum*	31,839	2,742.08	220.20	5.57	247.09
*Beta vulgaris*	29,386	7,504.30	233.77	9.78	321.39
*Spinacia oleracea*	38,319	6,762.69	252.04	8.86	335.22

**Fig. 5:**
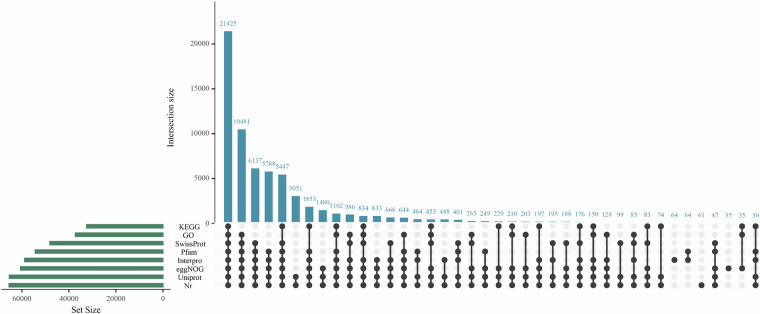
The UpSet plot of Gene function annotations. The intersection size of genes with functional annotation using multiple public databases.

Gene functions were assigned aligned all predicted protein-coding genes against multiple publicly available databases such as Nr (http://www.ncbi.nlm.nih.gov/protein/), Uniprot, InterPro, Pfam, Swissprot, GO, and KEGG. Overall, 65,774 protein-coding genes were functionally annotated in at least one database (Fig. 5, Table 9). Among these annotated genes, 65,441 genes were annotated in the Nr database^36^, 65,312 genes were annotated in the Uniport database^37^, 58,797 genes were annotated in the InterPro database^38^, 54,309 genes were annotated in the Pfam database^39^, 48,078 in the Swiss-Prot database^40^, 37,217 in the GO database^41^, and 32,456 in the KEGG database^42^ (Fig. 5, Table 9).

**Table 9:** Summary of gene function annotations.

Categories	Annotated gene number	Percent (%)
Annotated	65,774	95.82%
Nr	65,441	95.33%
Uniport	65,312	95.14%
InterPro	58,797	85.65%
Pfam	54,309	79.11%
Swissprot	48,078	70.04%
GO	37,217	54.22%
KEGG	32,456	47.28%
Unannotated	2,872	4.18%

## Data Records

The raw sequence data reported in this paper have been deposited in the Genome Sequence Archive (GSA^43^) in National Genomics Data Center^44^. Access to this data is available to the public under the accession number PRJCA030379, which can be found with the following GSA IDs: CRA019251^45^, CRA019182^46^, CRA019183^47^, CRA019451^48^. The assembled genome sequence has been made available in GenBank with JBLJBX000000000^49^. Additionally, the annotation data has been deposited at the Figshare repository^50^.

## Technical Validation

DNA quality was assessed using 1% agarose gel electrophoresis, and DNA concentration was measured with Qubit 3.0 Fluorometer, achieving an absorbance ratio of around 2.0 at 260/280. We used Fastp^14^ to assess the quality scores of all bases in the raw sequencing data. Additionally, the 17-mer distribution analysis was performed on the clean data to estimate the target genome size. The genome size estimated by the survey closely matched the assembled genome size, further supporting the reliability of the evaluation results.

The genome-wide Hi-C interaction heatmap was generated using Juicerbox. In the heatmap, the coordinates represent bins across individual chromosomes, with the color of each point reflecting the logarithmic value of the interaction strength between corresponding bin pairs (Fig. 4). Notably, regions with higher interaction strength are represented by deeper colors, and the diagonal shows significantly stronger interactions compared to the ends.

The scaffold N50, the length at which half of the genome assembly is represented in scaffolds of that size, improved significantly to 158.33 Mb, indicating high assembly quality (Table 3). For the genome evaluation, 95.20% of BUSCOs were classified as complete, with 19.20% being single-copy and 76.00% being duplicated. Fragmented BUSCOs made up only 1.30%, while 3.50% were missing. The gene set evaluation similarly shows a high percentage of completeness at 94.30%, with 22.80% single-copy and 71.50% duplicated BUSCOs. Fragmented BUSCOs were slightly lower at 0.60%, and missing BUSCOs were higher at 5.10%. The BUSCO analysis indicates excellent sequencing quality, with over 94% of BUSCOs complete in both the genome and gene set, suggesting minimal fragmentation and high completeness in the assembly. The presence of a higher proportion of duplicated BUSCOs may indicate some degree of redundancy, but the low percentage of missing and fragmented BUSCOs further confirms the robustness of the assembly (Table 5).

## Usage Notes

The final assembled *R. japonica* genome spans approximately 3.30 Gb, larger than the 2.56 Gb genome of *P. cuspidatum*^11^. Although both genomes contain a high proportion of repetitive sequences, *R. japonica* has a slightly higher percentage (74.79% compared to 71.54%). However, *R. japonica* exhibits superior assembly quality, with an N50 of 1.39 Mb, and 99.22% of the sequences are anchored to 22 pseudo-chromosomes, demonstrating a high level of assembly integrity. Future research could explore gene functions in *R. japonica* that are linked to its invasiveness and pharmacological properties, as well as utilize this reference genome for selective breeding initiatives.

## Data Availability

All software and pipelines were executed following the manuals and protocols of the published bioinformatics tools. The software versions and parameters are detailed in the Methods section. No custom programming or coding was used.
